# Using Machine-Learning to Assess the Prognostic Value of Early Enteral Feeding Intolerance in Critically Ill Patients: A Retrospective Study

**DOI:** 10.3390/nu15122705

**Published:** 2023-06-10

**Authors:** Orit Raphaeli, Liran Statlender, Chen Hajaj, Itai Bendavid, Anat Goldstein, Eyal Robinson, Pierre Singer

**Affiliations:** 1 Industrial Engineering and Management, Ariel University, Ariel 40700, Israel; chenha@ariel.ac.il (C.H.); anatgo@ariel.ac.il (A.G.); 2 Institute for Nutrition Research, Beilinson Hospital, Rabin Medical Center, Petah Tikva 4941492, Israel; psinger@clalit.org.il; 3 Data Science and Artificial Intelligence Research Center, Ariel University, Ariel 40700, Israel; 4 Intensive Care Unit, Beilinson Hospital, Rabin Medical Center, Petah Tikva 4941492, Israel; liranst1@clalit.org.il (L.S.); itaibd@clalit.org.il (I.B.); eyalrob@clalit.org.il (E.R.)

**Keywords:** enteral nutrition, gastrointestinal intolerance, enteral feeding intolerance, machine learning, prediction models

## Abstract

Background: The association between gastrointestinal intolerance during early enteral nutrition (EN) and adverse clinical outcomes in critically ill patients is controversial. We aimed to assess the prognostic value of enteral feeding intolerance (EFI) markers during early ICU stays and to predict early EN failure using a machine learning (ML) approach. Methods: We performed a retrospective analysis of data from adult patients admitted to Beilinson Hospital ICU between January 2011 and December 2018 for more than 48 h and received EN. Clinical data, including demographics, severity scores, EFI markers, and medications, along with 72 h after admission, were analyzed by ML algorithms. Prediction performance was assessed by the area under the receiver operating characteristics (AUCROC) of a ten-fold cross-validation set. Results: The datasets comprised 1584 patients. The means of the cross-validation AUCROCs for 90-day mortality and early EN failure were 0.73 (95% CI 0.71–0.75) and 0.71 (95% CI 0.67–0.74), respectively. Gastric residual volume above 250 mL on the second day was an important component of both prediction models. Conclusions: ML underlined the EFI markers that predict poor 90-day outcomes and early EN failure and supports early recognition of at-risk patients. Results have to be confirmed in further prospective and external validation studies.

## 1. Introduction

Early enteral feeding (EN) has been recommended by many international societies [1,2,3] in patients admitted to the intensive care unit (ICU). However, reaching energy and protein targets may be hindered by gastrointestinal (GI) intolerance [4,5]. GI intolerance—also referred to as enteral feeding intolerance (EFI)—is defined as features of GI dysfunction appearing during EN [6]. This condition increases the energy deficit and may be deleterious to the patient [7]. EFI is a common problem in the ICU, characterized by a multifaceted clinical representation of markers including large gastric residual volumes (GRVs), GI symptoms, and inadequate delivery of EN [8]. The use of the number of GI symptoms as an additional component of the SOFA score in assessing multi-organ failure did not improve the accuracy of mortality prediction; however, using specific markers of EFI for this purpose has not been tested [9]. There are varying associations of EFI markers with outcomes, and it is not yet clear what the role of each marker is in predicting adverse outcomes [10,11]. This variation may stem from both testing different markers and the wide spectrum of pathophysiological mechanisms that affect different parts of the GI tract.

The management of EFI is somewhat controversial, and practices may vary [12]. This is evident when comparing the ESPEN and ASPEN societies’ recommendations. While ESPEN suggests considering supplemental parenteral nutrition (PN) in days 3–7 from ICU admission, ASPEN proposes to wait for 7–10 days to do so. As cited in [3], “they suggest that, in the patient at low nutrition risk (for example, NRS-2002 ≤ 3 or NUTR IC score ≤ 5), exclusive parenteral nutrition should be withheld over the first seven days following ICU admission if the patient cannot maintain volitional intake and if early EN is not feasible”. In addition, they recommend that “in patients at either low or high nutrition risk, use of supplemental PN should be considered after 7–10 days if unable to meet more than 60% of energy and protein requirements by the enteral route alone. Initiating supplemental PN prior to this 7–10-days period in critically ill patients on some EN does not improve outcomes and may be detrimental to the patient”. However, many patients do not reach 60% of their prescription. Several interruptions, including under-prescription but also gastrointestinal intolerance, can explain these findings [13].

Recently, novel machine learning (ML) techniques have demonstrated improved predictive performance in the medical domain in modeling complex and non-linear effects compared to standard statistical prediction models [14]. In the domain of critical care medicine, the number of publications using ML models has increased rapidly. The most common aims of these studies include predicting mortality, predicting complications, and improving prognostic models [15,16]. For example, a recent systematic review demonstrated that ML models can accurately predict the onset of sepsis in ICU patients, outperforming traditional scoring tools [17]. In the area of clinical nutrition, few studies have used the ML approach to develop predictive models of mortality [18] or feeding intolerance [19]. The ML approach has not yet been used to assess the prognostic value of EFI markers in the early acute phase, nor has it been used for the early prediction of enteral feeding failure.

In an attempt to evaluate the risks associated with early EFI and to predict the failure of enteral feeding without waiting a week, the aim of our study was to use the ML approach to find the prognostic value of EFI markers and to predict early EN failure, supporting the decision-making of the practitioner in this challenging task.

## 2. Materials and Methods

### 2.1. Study Design and Setting

The study was designed as a retrospective cohort study using electronic health record data of patients admitted to the general ICU at Beilinson Hospital, Rabin Medical Center, Israel, between January 2011 and December 2018. The general ICU has 16 beds, admitting more than six hundred patients annually. The study was approved by the Ethical Review Board of Rabin Medical Center (approval number: RMC 0392-14) and performed in accordance with the Declaration of Helsinki.

### 2.2. Data Sources

The data was extracted from two computerized database systems of electronic health records (EHR): the ICU EHR (iMD-soft Metavision) and the hospital EHR (Chameleon). The ICU database was used to extract patient data such as admission details, GI symptoms, nutritional treatment, and medications, while the hospital database was used to extract the death registry. All extracted records were de-identified for the study. Our study followed the Transparent Reporting of a Multivariable Prediction Model for Individual Prognosis or Diagnosis (TRIPOD) reporting guidelines for prediction model development and validation [20].

### 2.3. Population

The study population included all adult patients who stayed in the ICU for more than 48 h and received EN. Patients who received exclusive PN as well as patients who received oral feeding, or no feeding were excluded. Patients with more than 20% missing values were excluded as well. For patients with multiple encounters, we included only the first encounter, while the remaining encounters were excluded.

### 2.4. Features

The features included four broad classes of EHR data measured over 72 h following ICU admission, including demographic characteristics, severity scores, EFI markers, and medications applied to manage EFI.

#### Features Definitions

1. Demographic characteristics included age, gender, BMI, and admission type;
2. Severity scores included the APACHEII score and the SOFA score on day one and day three (0–24 h and 48–72 h after ICU admission, respectively);
3. EFI markers were defined according to three common categories identified in a systematic review [8], including “large” gastric residual volumes (GRVs), GI symptoms, and “inadequate” delivery of EN. We included daily level markers under each category. Makers were preprocessed, using the following transformations:
  - Daily ‘large’ GRV was defined according to a threshold of 250 mL, which was found to be the median value used in previous studies (ranging between 150 and 500 mL) [8]. The feature GRV on day x (x = 1–3) was coded into a dichotomous variable that denotes the occurrence/non-occurrence of a GRV amount greater than 250 mL in 24 h;
  - Daily GI symptoms were defined according to established definitions [9]: Daily vomiting/regurgitation was defined as visible vomiting or regurgitation in any amount in 24 h. Daily diarrhea was defined as loose or liquid stool three or more times within 24 h. Daily GI bleeding was defined as the visible appearance of blood in vomitus, nasogastric aspirate, or stool in 24 h. The feature GI symptom on day x (x = 1–3) was coded into a dichotomous feature denoting the occurrence/non-occurrence of a symptom in 24 h;
  - Inadequate delivery of EN was defined according to the proportion of ‘energy requirements’ delivered on day three [8], with a cutoff of energy administration below 70% of the defined target [1]. The feature of inadequate delivery of EN on day three was coded into a dichotomous feature denoting an administration of less than 70% of the calculated caloric needs.
4. Medications applied to manage EFI refer to prokinetic agents. Daily prokinetic agent usage was defined according to the daily intake of metoclopramide and erythromycin. Prokinetic agent on day x (x = 1–3) was coded into a dichotomous feature denoting the use/no use of these medications in 24 h.

### 2.5. Missing Data

No imputation was made. Only full records were included.

### 2.6. Outcomes

Ninety-day mortality was defined as the primary outcome. ICU, hospital, and 28-day mortality, as well as prolonged ICU length of stay (LOS)—defined as more than seven days of ICU stay—were defined as secondary outcomes.

### 2.7. Prediction of Early EN Failure

To develop a prediction model of early EN failure, we defined the outcome as inadequate delivery of EN on day three and used features from days 1–2 as predictors. The features included demographic features, severity scores, large GRV, GI symptoms, and prokinetic usage.

### 2.8. Statistical Analysis

Data were expressed as frequencies (percentages) for categorical variables and as means ± SD or median (IQR) based on the test of normality for continuous variables. The Kolmogorov–Smirnov test was applied to test normality. Differences between the survivor and non-survivor groups were analyzed using Student’s *t*-test for continuous variables and Fisher’s exact test for categorical variables. The Mann–Whitney U test was used for the variable, which is not normally distributed. A correlation heatmap was used to test potential multicollinearity between features.

### 2.9. Model Development

The dataset was split using ten-fold cross-validation. Cross-validation is a method for evaluating the performance of a machine learning model by dividing the data into different train-test sets. This method helps prevent overfitting (i.e., when a model performs well on the training data but poorly on new, unseen data), and validate the generalization performance of the model. Using a ten-fold cross-validation approach, we divided the patient data in a stratified way into ten folds and conducted ten experiments. In the *i*-th (*i* = 1…10) experiment, we used the *i*-th fold of data as the test set and the remaining data as the training set. Candidate supervised learning classifiers—including K-nearest neighbor, decision tree, random forest, extreme gradient boosting (XGBoost), gradient boosting, logistic regression, and Ada-boost—were trained and evaluated. The mean value of the results of all ten experiments was computed to measure the performance of each classifier in terms of accuracy, precision, recall, specificity, F1 score (the harmonic mean of precision and recall), and the area under the curve of the receiver operating characteristic curve (AUCROC). The main performance measure in this study was AUCROC.

### 2.10. Model Interpretation

To explain the model, we used SHAP values to quantify the importance of each variable. SHAP values, which are based on the Shapely value from conditional game theory, are consistent and accurate calculations of the contributions of each feature to any ML model’s prediction [21].

### 2.11. Software

The modeling and statistical analysis were performed using IBM SPSS Statistics (Version 27) and the Scikit Learn library version 1.1, Python programming software version 3.7 (Python Programming Foundation, https://www.python.org/, (accessed on 1 January 2023)).

## 3. Results

### 3.1. Cohort Selection

**Figure 1:**
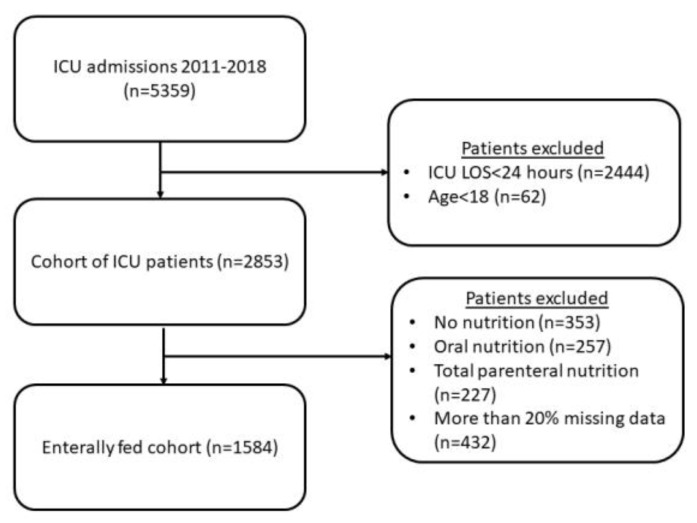
Study cohort.

During the study period, there were 5359 ICU admissions. We excluded 2444 patients whose ICU stay was less than 48 h and 62 patients whose age was less than 18. From these 2853 patients, we excluded 353 patients who received no nutrition, 257 patients who received oral nutrition, and 227 patients who received total parenteral nutrition. In 2016, we dropped 432 enterally fed patients with more than 20% missing data (Figure 1).

### 3.2. Cohort Characteristics

A total of 1584 patients were included in the study, of whom 612 (38.6%) died within 90 days of admission. The median LOS was 9.5 days (IQR: 5–19 days). The mean age of the patients was 59 years (SD = 17.5 years), with 64% being male. The median APACHEII score was 23 (IQR: 18–28), and the median SOFA scores on day one and day three were both 8 (IQR: 5–11). The occurrence rate of daily GI symptoms on days 1–3 was low, with 3.6–7.7% of patients having at least one episode of diarrhea, and less than 3% of patients having at least one episode of GI bleeding and vomiting. The occurrence rate of large GRV (>250 mL at least once in 24 h) on days 1–3 was between 21.5% and 24.2%. 64.3% of patients did not reach 70% of the caloric target on day 3. Metoclopramide and erythromycin intake rates on days 1–3 were 14–31% and less than 3%, respectively. We compared 90-day survivors to 90-day non-survivors. Compared with survivors, the non-survivor group was more likely to have diarrhea on day one (6.2% vs. 2.6%, *p* < 0.01), had higher rates of GRV > 250 mL on days 1–3 (27.9% vs. 21.8%, 27.8% vs. 16.9%, and 28.8% vs. 16.9%, respectively, *p* < 0.01), and had higher rates of erythromycin intake on days 2–3 (3.7% vs. 1.2%, 4.3% vs. 1.8%, *p* < 0.01). All variables are described in Table 1.

### 3.3. Correlation between Features and 90-Day Mortality

We examined correlations between features on days 1–3 and 90-day mortality using a correlation heatmap (Figure S1). As depicted in the heatmap, APACHEII, age, SOFA on the first and third days, and GRV on the second and third days had the highest correlation with 90-day mortality. Moreover, the plot revealed that there is no correlation between different EFI markers while there is a correlation between the occurrence of EFI markers and prokinetic intake during days one to three (e.g., diarrhea in days 1–3, metorphamide on days 1–3, etc.).

### 3.4. Prediction of 90-Day Mortality

We compared the performance of ML models to predict 90-day mortality using ROC analysis. The AUCROC of a gradient boosting model had the highest predictive value for 90-day mortality (AUCROC: 0.73; 95% CI 0.71–0.75), followed by a random forest model (AUCROC: 0.71; 95% CI 0.69–0.73) and logistic regression (AUCROC: 0.71; 95% CI 0.67—0.75). Moreover, the gradient boosting model achieved improved performance compared to a baseline comparator of a logistic regression model using the APACHEII score only. Other performance metrics show similar results. The detailed performance measures for all models are provided in Table 2. As shown in Table 2, the gradient boosting model achieved a recall of 0.54 ± 0.04, a specificity of 0.77 ± 0.04, and an F1 score of 0.58 ± 0.03.

**Figure 2:**
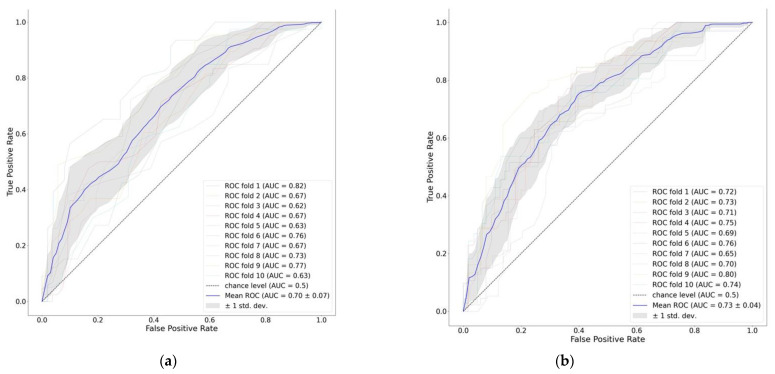
ROC curve of 90-day mortality prediction models: (**a**) APACHEII score; (**b**) features on days 1–3.

Figure 2 demonstrates the mean ROC of the gradient boosting model as well as the ROC of each fold of the ten-fold cross-validation.

**Figure 3:**
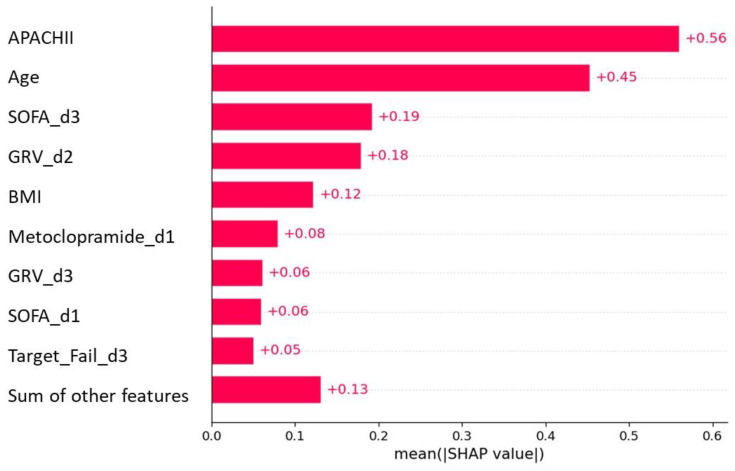
Feature importance plot of the 90-day mortality model.

To provide clinicians with a straightforward understanding of key variables related to predicting 90-day mortality, we categorized the top features of the best predictive model. This analysis of feature importance revealed that the APACHEII, age, and SOFA on the third day were the top predictors, followed by GRV > 250 mL on days 2–3, BMI, metoclopramide intake on day one, and inadequate delivery of EN on day 3 (Figure 3).

### 3.5. Prediction of Secondary Outcomes

We compared the performance of ML models to predict ICU, hospital, and 28-day mortality, as well as prolonged ICU stays (LOS > 7 days). For all secondary outcomes, the gradient-boosting classifier revealed the highest AUCROC. The ICU mortality prediction model revealed a higher AUCROC compared to the baseline comparator (0.73; 95% CI 0.69–0.76 vs. 0.70; 95% CI 0.64–0.75, respectively). The hospital mortality prediction model revealed a higher AUCROC compared to the baseline comparator (0.72; 95% CI 0.70–0.74 vs. 0.70; 95% CI 0.66–0.73, respectively). The 28-day mortality prediction model revealed a higher AUCROC compared to the baseline comparator (0.71; 95% CI 0.69–0.72 vs. 0.69; 95% CI 0.66–0.71, respectively). The prolonged LOS model revealed a low AUCROC. Yet, it was higher compared to the baseline comparator (0.57; 95% CI 0.54–0.59 vs. 0.52; 95% CI 0.47–0.56, respectively). The detailed performance measures of all models are shown in Supplementary Tables S2–S4. As shown in tables, 28-day hospital and ICU gradient-boosting mortality models achieved high specificity of 0.87 ± 0.03, 0.8 ± 0.02, 0.93 ± 0.02, recall of 0.3 ± 0.06, 0.47 ± 0.04, 0.29 ± 0.07, and F1 score of 0.37 ± 0.06, 0.51 ± 0.03, 0.37 ± 0.07, respectively. The long ICU prediction model achieved improved recall of 0.86 ± 0.02, specificity of 0.18 ± 0.03, and an F1 score of 0.76 ± 0.02.

### 3.6. Prediction of Early EN Failure

**Figure 4:**
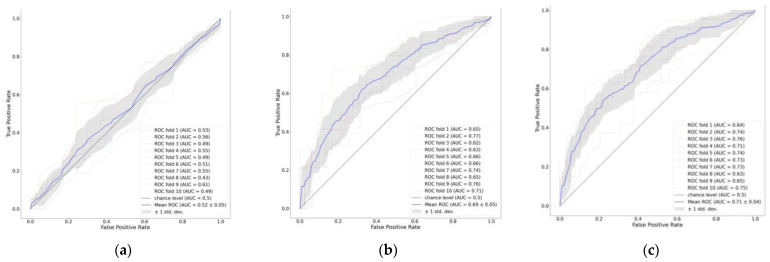
ROC curve of early EN failure prediction models. Predictors: (**a**) APACHEII score; (**b**) features on day 1; (**c**) features on days 1–2.

We compared ML models’ performance to predict inadequate EN delivery on day three based on features from days 1/1–2 using ROC analysis. The AUCROC of a gradient boosting model using features on day one was the highest among other ML models tested (AUCROC: 0.69; 95% CI 0.65–0.72). Adding features from the second day improved the results. The gradient boosting model using features on days 1–2 had the highest predictive value (AUCROC: 0.71; 95% CI 0.67–0.74). As shown in Figure 4, both models outperformed the baseline comparator of a logistic regression model with an APACHEII score only (AUCROC: 0.52; 95% CI 0.48–0.55). The detailed AUCROC and other performance measures for all models are provided in Supplementary Table S5.

**Figure 5:**
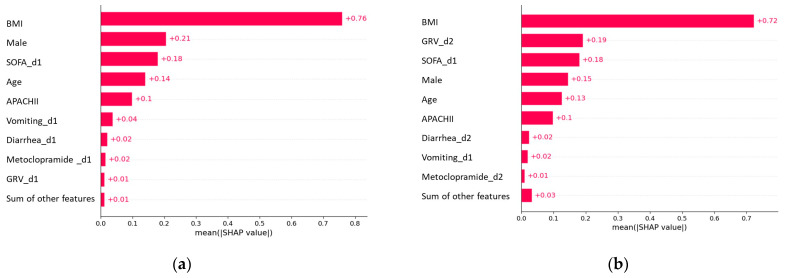
Feature importance plot of early EN failure prediction models. Predictors: (**a**) features on day 1; (**b**) features on days 1–2.

We used a feature importance plot to illustrate how predictors contribute to predicting early EN failure (Figure 5). The plot indicated that using features from day one to model EFI markers, such as vomiting, diarrhea, and GRV, on day one had a negligible contribution, while BMI, male, and SOFA on day one had the highest contribution (Figure 5a). Contrary, when using features from days 1–2, a large GRV on day two had a considerable contribution, after BMI, followed by SOFA on day 1. Other EFI markers, such as vomiting and diarrhea, had negligible contributions (Figure 5b).

## 4. Discussion

Early enteral feeding progression to target is often impaired by feeding interruptions and gastrointestinal intolerance [22]. This failure to reach the goal may be associated with an energy/protein deficit, increased complications, and even mortality [23]. Our study first confirms the importance of the gastric residual volume as a predictor of survival in critically ill patients. Following features such as APACHE II, age, or SOFA scores that have been largely evaluated and confirmed as relevant predictors of survival, GRV on days 2–3, and inadequate enteral feeding on day 3 are also relevant predictors of mortality, albeit they are less influential. These findings confirm the importance of GI intolerance and specifically the evaluation of the GRV in predicting the severity of the condition in ICU patients. This finding has been described previously in several large studies [4,5]. Interestingly, diarrhea and the number of defecations during the 3 days prior to sampling a positive blood culture (feature importance 0.008187, OR 0.34) have been found to be of significant importance in the prognosis of patients suffering from sepsis [24]. In our analysis, diarrhea is also highly ranked as a predictor of outcome in early enteral feeding and failure of enteral nutrition. Of these markers, GRV on day 2 has a relatively large contribution to the prediction of 90-day mortality, comparable to SOFA on day 3. While the individual SOFA score on day 3 is not a validated predictor of mortality, it has been shown that an increase or no change in SOFA after 48 h is associated with increased mortality [25]. Our use of ML shows which markers of EFI might provide a better assessment of GI failure as an organ system; further research is needed to assess this.

Our study further explores predictors of successful enteral feeding. This clinical daily dilemma leads to variable recommendations according to different guidelines, allowing the introduction of early supplemental parenteral nutrition or recommending observation and waiting up to 7–10 days to introduce supplemental parenteral nutrition. In our study, features such as BMI, SOFA on day 1, age, and gastric residual volume on day 2 were predictors of enteral feeding failure and should be considered when deciding whether to continue to prescribe enteral feeding at an increasing level, at a stabilizing rate, or to utilize parenteral nutrition, whether supplemental or total. Further studies may be helpful to support such decisions. However, since the same recommendations might not suit all patients (i.e., “one size does not fit all”), an ML approach may assist the treating physician in deciding how to handle gastrointestinal intolerance in a critically ill setting. Other examples have been suggested in different fields. One example is an ML-based model to decide when to intubate a COVID-19 patient [26]. In the field of hydration and nutrition, ML was able to define, in addition to APACHE IV and post-surgery status, other predictive features of fluid overload, such as being at risk of malnutrition, elevated lactate and bicarbonate, and low creatinine [27]. Predicting refeeding syndrome was also explored [28]. Features such as admission to the ICU, recent weight loss, diabetes mellitus with insulin use, initial phosphorus level, and use of furosemide were recognized as having a major effect on the development of refeeding syndrome and may help health professionals with the early recognition of at-risk patients.

Limitations: Our study is a retrospective single-center study, and therefore, the proposed results have to be validated on data from other centers as well as prospectively. Yet, the ML approach requires the use of an electronic health recording system, and not all the centers are equipped with such computerized systems. Moreover, implementation of the models in other centers might require additional adjustments. In addition, the relative strength of the predictive values suggests that the inclusion of additional clinical data such as temporal lab tests and vital signs may improve models’ performance.

Strengths: In the ICU setting, where patient demographics, clinical characteristics, and organizational practices may differ, our machine learning approach can address the specific patient profile and utilize all available data and treatments. An ML approach may enhance the decision-making of healthcare professionals by considering not only guidelines and prospective randomized studies but also the individualized conditions of the patient, thus optimizing medical nutritional therapy throughout the entire hospitalization.

## 5. Conclusions

Machine learning underlined the GI intolerance markers that predict a poor 90-day outcome. GRV above 250 mL seems to be an important component of these predictors. In addition, it was possible to predict early enteral feeding failure in male patients suffering from high BMI, older age, a high SOFA score on day one, and a large gastric residual. Such a machine learning approach can serve as a support tool in the early acute phase for clinical nutrition decisions. While external validation studies are required, the findings of our study provide support for the utilization of machine learning algorithms to improve risk stratification among enterally fed critically ill patients.

## Figures and Tables

**Table 1:** Cohort characteristics.

Variable	All Patients (*n* = 1584)	Survivors (90-Day) (*n* = 972)	Non-Survivors (90-Day) (*n* = 612)	*p*-Value
age Mean ± SD	59.05 ± 17.5	55.8 ± 18.3	64.1 ± 14.7	0.00
gender (female) *n* (%)	590 (37%)	372 (38.3%)	218 (35.6%)	ns
BMI Median [IQR]	26.5 [23.5, 30.8]	26.4 [23.4, 30.8]	26.6 [23.8, 30.6]	ns
Severity scores on day one and day three Median [IQR]				
APACHEII	23 [18, 28]	21 [16, 26]	26 [21, 31]	0.00
SOFA-d1	8 [5, 11]	7 [4, 10]	9 [7, 11]	0.00
SOFA-d3	8 [5, 11]	7 [4, 10]	10 [7, 13]	0.00
Daily GI symptoms on days 1–3 *n* (%)				
Diarrhea-d1	57 (3.6%)	25 (2.6%)	32 (6.2%)	0.006
Diarrhea-d2	80 (5.1%)	43 (4.4%)	37 (6%)	ns
Diarrhea-d3	122 (7.7%)	69 (7.1%)	53 (8.7%)	ns
GI bleeding-d1	28 (1.8%)	14 (1.4%)	14 (2.3%)	ns
GI bleeding-d2	27 (1.7%)	15 (1.5%)	12 (1.9%)	ns
GI bleeding-d3	24 (1.5%)	15 (1.5%)	9 (1.5%)	ns
Vomiting-d1	48 (3%)	30 (3.1%)	18 (2.9%)	ns
Vomiting-d2	40 (2.5%)	26 (2.7%)	14 (2.3%)	ns
Vomiting-d3	47 (3%)	33 (3.4%)	14 (2.3%)	ns
Daily large gastric residual volume on days 1–3 *n* (%)				
GRV > 250-d1	383 (24.2%)	212 (21.8%)	171 (27.9%)	0.006
GRV > 250-d2	334 (21.1%)	164 (16.9%)	170 (27.8%)	0.00
GRV > 250-d3	340 (21.5%)	164 (16.9%)	176 (28.8%)	0.00
Inadequate delivery of EN on day three *n* (%)				
Target Fail-d3 (<70%)	1019 (64.3%)	630 (65.1%)	389 (63.7%)	ns
Daily prokinetic intake on days 1–3 *n* (%)				
Metoclopramide-d1	207 (14%)	132 (14.6%)	75 (13.2%)	ns
Metoclopramide-d2	371 (27%)	225 (27.3%)	146 (26.7%)	ns
Metoclopramide-d3	392 (30.9%)	231 (31.3%)	161 (30.7%)	ns
Erythromycin-d1	17(1.2%)	10 (1.1%)	7 (1.2%)	ns
Erythromycin-d2	30 (2.2%)	10(1.2%)	20 (3.7%)	0.002
Erythromycin-d3	36 (2.8%)	13 (1.8%)	23 (4.3%)	0.007
Clinical outcomes				
LOS Median [IQR]	9.5 [5, 19]	9 [5, 19]	10 [6, 18]	ns
Prolonged LOS (LOS > 7 d)	952 (60.1%)	556 (57.2%)	396 (64.7%)	0.003
90-day mortality *n* (%)	612 (38.6%)			
28-day mortality *n* (%)	444 (28%)			
ICU mortality *n* (%)	358 (22.6%			
Hospital mortality *n* (%)	518 (32.7%)			

ns—non significant.

**Table 2:** Predictive performance of the 90-day prediction models.

	Accuracy (SD)	Precision (SD)	Recall (SD)	Specificity (SD)	F1 (SD)	AUCROC (95% CI)
Features on Days 1–3						
Gradient Boosting Classifier	0.68 (0.03)	0.63 (0.06)	0.54 (0.04)	0.77 (0.04)	0.58 (0.05)	0.73 (0.71–0.75)
Random Forest Classifier	0.66 (0.03)	0.61 (0.06)	0.53 (0.05)	0.75 (0.04)	0.57 (0.04)	0.71 (0.69–0.73)
Logistic Regression	0.66 (0.04)	0.62 (0.04)	0.53 (0.05)	0.76 (0.04)	0.57 (0.04)	0.71 (0.67–0.75)
AdaBoost Classifier	0.64 (0.04)	0.59 (0.09)	0.53 (0.06)	0.72 (0.08)	0.55 (0.05)	0.69 (0.65–0.73)
XGB Classifier	0.63 (0.03)	0.57 (0.06)	0.54 (0.04)	0.71 (0.06)	0.55 (0.04)	0.69 (0.66–0.72)
KN neighbors Classifier	0.65 (0.03)	0.59 (0.05)	0.52 (0.04)	0.74 (0.03)	0.55 (0.04)	0.68 (0.66–0.70)
Decision Tree Classifier	0.60 (0.02)	0.52 (0.05)	0.51 (0.05)	0.66 (0.02)	0.51 (0.04)	0.58 (0.56–0.60)
Baseline comparator: APACHE II						
Logistic Regression	0.65 (0.03)	0.62 (0.09)	0.45 (0.05)	0.76 (0.06)	0.52 (0.05)	0.70 (0.66–0.74)

## Data Availability

Data and materials will be available upon request according to the rules of Clalit Health Care and the Rabin Medical Center.

## Supplementary Materials

The following supporting information can be downloaded at: https://www.mdpi.com/article/10.3390/nu15122705/s1, Figure S1: correlation heatmap between study features and 90-day mortality; Table S1: Predictive performance of 28-day mortality models; Table S2: Predictive performance of hospital mortality models; Table S3: Predictive performance of ICU mortality models; Table S4: Predictive performance of long ICU stay (ICU-LOS > 7 days) models; Table S5: Predictive performance of early EN failure.
