# Supplementary material for: Using Machine-Learning to Assess the Prognostic Value of Early Enteral Feeding Intolerance in Critically Ill Patients: A Retrospective Study
Source: Nutrients. 2023 Jun 10;15(12):2705. doi: 10.3390/nu15122705 (PMC10305247; doi:10.3390/nu15122705)
Supplement: Supplementary file 1 [file nutrients-15-02705-s001.zip › nutrients-2375682-supplementary.pdf]

Using Machine-Learning to Assess the Prognostic Value of Early Enteral Feeding Intolerance in Critically Ill Patients: A Retrospective Study

Orit Raphaeli [1,2,3,*], Liran Statlender [4], Chen Hajaj [1,3], Itai Bendavid [4], Anat Goldstein [1,3], Eyal Robinson [4] and Pierre Singer [2,4]

[1] Industrial Engineering and Management, Ariel University, Ariel 40700, Israel
[2] Institute for Nutrition Research, Beilinson Hospital, Rabin Medical Center, Petah Tikva 4941492, Israel
[3] Data Science and Artificial Intelligence Research Center, Ariel University, Ariel 40700, Israel
[4] Intensive Care Unit, Beilinson Hospital, Rabin Medical Center, Petah Tikva 4941492, Israel
* Correspondence: oritr@ariel.ac.il; Tel.: +972-546730795

Supplementary materials

Supplementary material 1. Correlation heatmap

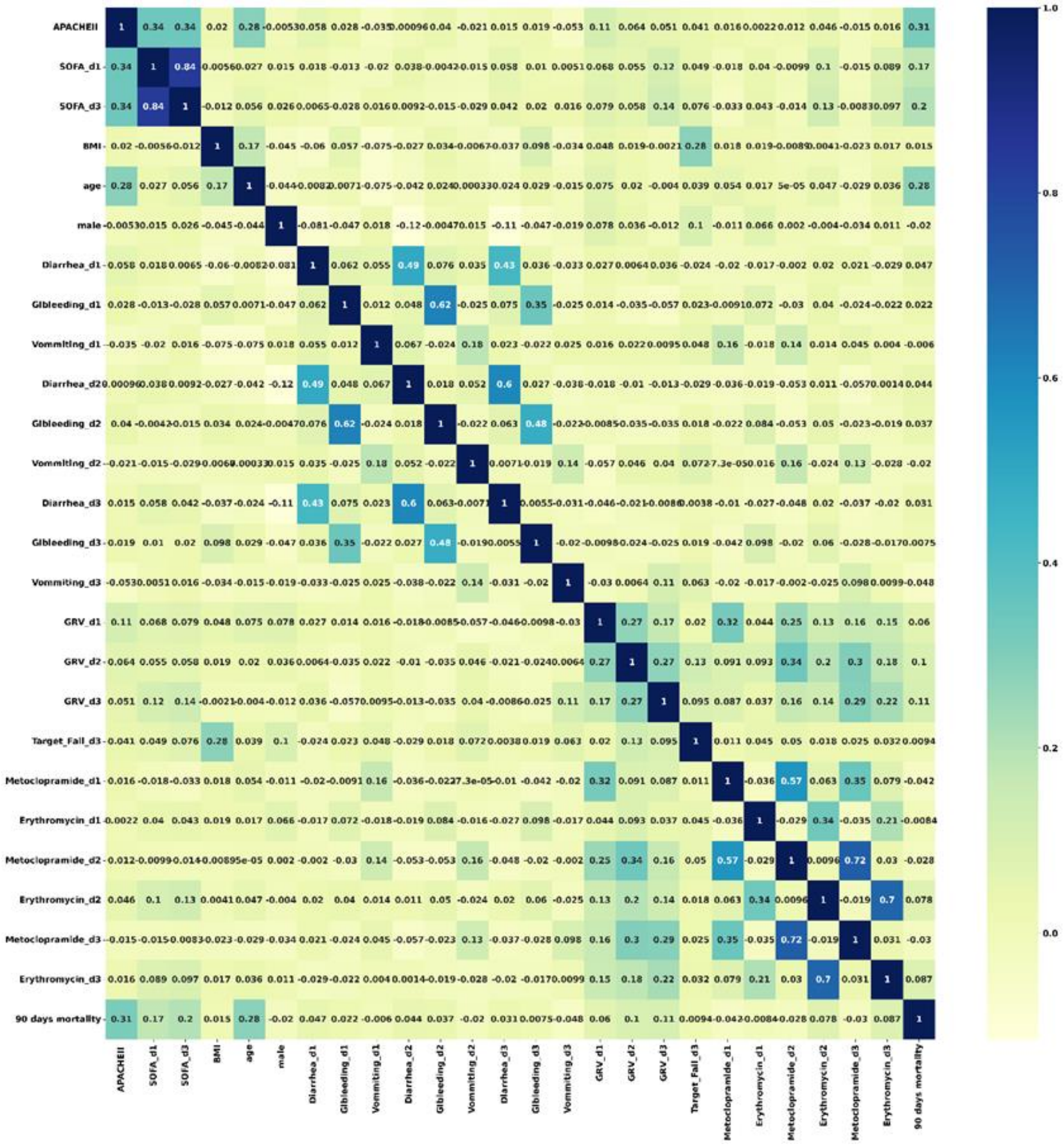

Figure S1. Correlation heatmap between study features and 90-day mortality

Supplementary material 2. Predictive performance of secondary outcomes

Table S1: Predictive performance of 28-day mortality models

	Accuracy (SD)	Precision (SD)	Recall (SD)	Specificity (SD)	F1 (SD)	AUCROC (95% CI)
**Features within 72 h after ICU admission**						
Gradient boosting Classifier	0.70 (0.02)	0.50 (0.09)	0.30 (0.06)	0.87 (0.03)	0.37 (0.06)	0.71 (0.69-0.72)
Logistic Regression	0.73 (0.03)	0.59 (0.11)	0.31 (0.06)	0.90 (0.03)	0.40 (0.07)	0.71 (0.67-0.74)
Random Forest Classifier	0.71 (0.02)	0.51 (0.06)	0.30 (0.07)	0.88 (0.02)	0.37 (0.07)	0.69 (0.66-0.71)
AdaBoost Classifier	0.70 (0.04)	0.50 (0.07)	0.36 (0.07)	0.85 (0.03)	0.41 (0.06)	0.67 (0.62-0.71)
KN neighbors Classifier	0.69 (0.03)	0.48 (0.09)	0.35 (0.07)	0.84 (0.02)	0.40 (0.07)	0.67 (0.63-0.70)
XGB Classifier	0.68 (0.03)	0.47 (0.05)	0.39 (0.06)	0.81 (0.03)	0.42 (0.04)	0.66 (0.63-0.68)
Decision Tree Classifier	0.63 (0.04)	0.39 (0.07)	0.42 (0.07)	0.72 (0.03)	0.40 (0.07)	0.57 (0.52-0.61)
**Baseline comparator: APACHE II**						
Logistic Regression	0.70 (0.03)	0.57 (0.12)	0.14 (0.04)	0.75 (0.01)	0.22 (0.05)	0.69 (0.66-0.71)

Table S2: Predictive performance of hospital mortality models

	Accuracy (SD)	Precision (SD)	Recall (SD)	Specificity (SD)	F1 (SD)	AUCROC (95% CI)
**Features within 72 h after ICU admission**						
Gradient Boosting Classifier	0.68 (0.02)	0.57 (0.05)	0.47 (0.04)	0.80 (0.02)	0.51 (0.03)	0.72 (0.70-0.74)
Random Forest Classifier	0.69 (0.03)	0.60 (0.05)	0.44 (0.04)	0.83 (0.03)	0.51 (0.04)	0.72 (0.69-0.74)
Logistic Regression	0.68 (0.03)	0.58 (0.09)	0.39 (0.05)	0.84 (0.03)	0.47 (0.06)	0.72 (0.69-0.74)
AdaBoost Classifier	0.67 (0.02)	0.56 (0.07)	0.42 (0.03)	0.81 (0.04)	0.48 (0.03)	0.70 (0.66-0.73)
KN neighbors Classifier	0.67 (0.04)	0.55 (0.05)	0.43 (0.05)	0.80 (0.04)	0.48 (0.05)	0.68 (0.64-0.71)
XGB Classifier	0.66 (0.03)	0.53 (0.07)	0.45 (0.05)	0.78 (0.04)	0.48 (0.05)	0.67 (0.63-0.70)
Decision Tree Classifier	0.61 (0.03)	0.46 (0.06)	0.47 (0.04)	0.69 (0.04)	0.46 (0.04)	0.58 (0.55-0.60)
**Baseline comparator: APACHE II**						
Logistic Regression	0.69 (0.03)	0.62 (0.08)	0.33 (0.07)	0.88 (0.03)	0.42 (0.07)	0.70 (0.66-0.73)

Table S3: Predictive performance of ICU mortality models

	Accuracy (SD)	Precision (SD)	Recall (SD)	Specificity (SD)	F1 (SD)	AUCROC (95% CI)
**Features within 72 h after ICU admission**						
Gradient Boosting Classifier	0.77 (0.03)	0.57 (0.08)	0.29 (0.07)	0.93 (0.02)	0.37 (0.07)	0.73 (0.69-0.76)
Random Forest Classifier	0.77 (0.01)	0.57 (0.10)	0.22 (0.05)	0.94 (0.01)	0.31 (0.06)	0.72 (0.67-0.76)
Logistic Regression	0.78 (0.01)	0.61 (0.09)	0.23 (0.04)	0.95 (0.01)	0.33 (0.05)	0.72 (0.68-0.75)
AdaBoost Classifier	0.74 (0.03)	0.45 (0.07)	0.28 (0.06)	0.89 (0.02)	0.34 (0.06)	0.69 (0.64-0.73)
XGB Classifier	0.74 (0.02)	0.47 (0.07)	0.32 (0.06)	0.88 (0.02)	0.38 (0.06)	0.69 (0.65-0.72)
KN neighbors Classifier	0.74 (0.01)	0.43 (0.10)	0.25 (0.06)	0.89 (0.02)	0.31 (0.07)	0.66 (0.61-0.70)
Decision Tree Classifier	0.70 (0.03)	0.38 (0.05)	0.38 (0.04)	0.80 (0.03)	0.38 (0.04)	0.59 (0.56-0.61)
**Baseline comparator: APACHE II**						
Logistic Regression	0.76 (0.01)	0.60 (0.24)	0.07 (0.03)	0.78 (0.01)	0.13 (0.05)	0.70 (0.64-0.75)

Table S4: Predictive performance of long ICU stay (ICU-LOS>7 days) models

	Accuracy (SD)	Precision (SD)	Recall (SD)	Specificity (SD)	F1 (SD)	AUCROC (95% CI)
**Features within 72 h after ICU admission**						
Random Forest Classifier	0.63 (0.03)	0.67 (0.03)	0.86 (0.02)	0.18 (0.03)	0.76 (0.02)	0.57 (0.54-0.59)
Logistic Regression	0.66 (0.03)	0.67 (0.03)	0.95 (0.02)	0.09 (0.02)	0.79 (0.02)	0.57 (0.51-0.62)
Gradient Boosting Classifier	0.65 (0.02)	0.68 (0.03)	0.90 (0.02)	0.15 (0.03)	0.77 (0.02)	0.55 (0.51-0.58)
AdaBoost Classifier	0.64 (0.03)	0.67 (0.03)	0.88 (0.02)	0.16 (0.04)	0.76 (0.02)	0.54 (0.49-0.58)
XGB Classifier	0.62 (0.02)	0.69 (0.03)	0.78 (0.03)	0.30 (0.05)	0.73 (0.02)	0.54 (0.51-0.56)
Decision Tree Classifier	0.57 (0.02)	0.68 (0.02)	0.67 (0.02)	0.37 (0.03)	0.67 (0.02)	0.52 (0.49-0.54)
KN neighbors Classifier	0.60 (0.03)	0.66 (0.02)	0.79 (0.04)	0.21 (0.03)	0.72 (0.02)	0.49 (0.45-0.52)
**Baseline comparator: APACHE II**						
Logistic Regression	0.66 (0.02)	0.66 (0.02)	0.55 (0.06)	0.22 (0.03)	0.80 (0.02)	0.52 (0.47-0.56)

Supplementary material 3. Predictive performance of of early EN failure

Table S5: Predictive performance of early EN failure

	**Accuracy (SD)**	**Precision (SD)**	**Recall (SD)**	**Specificity (SD)**	**F1 (SD)**	**AUCROC (95% CI)**
**Features within 24 h after ICU admission**						
Gradient Boosting Classifier	0.67 (0.03)	0.69 (0.04)	0.88 (0.02)	0.29 (0.04)	0.77 (0.03)	0.69 (0.65-0.72)
Logistic Regression	0.67 (0.03)	0.71 (0.03)	0.80 (0.04)	0.42 (0.05)	0.75 (0.03)	0.69 (0.65-0.72)
AdaBoost Classifier	0.66 (0.04)	0.70 (0.04)	0.82 (0.02)	0.37 (0.06)	0.75 (0.03)	0.68 (0.64-0.71)
Random Forest Clas-sifier	0.65 (0.04)	0.71 (0.05)	0.79 (0.03)	0.42 (0.05)	0.74 (0.04)	0.67 (0.62-0.71)
XGB Classifier	0.63 (0.04)	0.70 (0.07)	0.75 (0.03)	0.42 (0.07)	0.72 (0.04)	0.64 (0.60-0.67)
Decision Tree Classi-fier	0.62 (0.03)	0.68 (0.04)	0.78 (0.02)	0.33 (0.06)	0.73 (0.03)	0.60 (0.56-0.63)
KN neighbors Classi-fier	0.60 (0.03)	0.69 (0.02)	0.70 (0.04)	0.43 (0.03)	0.69 (0.03)	0.56 (0.52-0.59)
**Features within 48 h after ICU admission**						
Gradient Boosting Classifier	0.69 (0.03)	0.73 (0.03)	0.82 (0.05)	0.46 (0.05)	0.77 (0.03)	0.71 (0.67-0.74)
Logistic Regression	0.68 (0.04)	0.71 (0.05)	0.87 (0.02)	0.36 (0.07)	0.78 (0.03)	0.71 (0.67-0.74)
Ada Boost Classifier	0.68 (0.03)	0.71 (0.04)	0.84 (0.03)	0.40 (0.03)	0.77 (0.03)	0.70 (0.66-0.73)
Random Forest Clas-sifier	0.66 (0.03)	0.70 (0.03)	0.82 (0.03)	0.38 (0.06)	0.75 (0.02)	0.68 (0.64-0.71)
XGB Classifier	0.64 (0.01)	0.70 (0.04)	0.75 (0.02)	0.44 (0.05)	0.73 (0.01)	0.65 (0.63-0.66)
KN neighbors Classi-fier	0.62 (0.02)	0.68 (0.03)	0.77 (0.04)	0.35 (0.05)	0.72 (0.02)	0.60 (0.55-0.64)
Decision Tree Classi-fier	0.59 (0.03)	0.68 (0.05)	0.67 (0.02)	0.44 (0.06)	0.67 (0.03)	0.56 (0.53-0.58)
**Baseline comparator: APACHE II**						
Logistic Regression	0.64 (0.03)	0.64 (0.03)	1.00 (nan))	0.00 (nan)	0.78 (0.02)	0.52 (0.48-0.55)
